# A Deeper Statistical Examination of Arrival Dates of Migratory Breeding Birds in Relation to Global Climate Change

**DOI:** 10.3390/biology2020742

**Published:** 2013-04-26

**Authors:** W. Herbert Wilson

**Affiliations:** Department of Biology, Colby College, 5739 Mayflower Hill Drive, Waterville, ME 04901, USA; E-Mail: whwilson@colby.edu; Tel.: +1-207-859-573

**Keywords:** birds, migration, skewness, kurtosis, arrival dates, weather, climate change, phenology

## Abstract

Using an 18-year dataset of arrival dates of 65 species of Maine migratory breeding birds, I take a deeper view of the data to ask questions about the shapes of the distribution. For each year, most species show a consistent right-skewed pattern of distribution, suggesting that selection is stronger against individuals that arrive too early compared to those that arrive later. Distributions are consistently leptokurtic, indicating a narrow window of optimal arrival dates. Species that arrive earlier in the spring show higher skewness and kurtosis values. Nectarivorous species showed more pronounced skewness. Wintering area did not explain patterns of skewness or kurtosis. Deviations from average temperatures and the North Atlantic Oscillation index explained little variation in skewness and kurtosis. When arrival date distributions are broken down into different medians (e.g., 5% median and 75% median), stronger correlations emerge for portions of the distribution that are adjacent, suggesting species fine-tune the progress of their migration. Interspecific correlations for birds arriving around the same time are stronger for earliest migrants (the 25% median) compared to the true median and the 75% median.

## 1. Introduction

Abundant evidence for global climate change exists in monotonic increases in carbon dioxide concentrations, melting of polar ice caps with concomitant sea level rise, and record hot temperatures around the world [1,2,3,4]. Phenological data add to this body of evidence [5,6,7,8,9,10,11,12,13]. Changing migration schedules of birds provide some of the most compelling indications that these climatic changes are influencing the natural world. Birds are unique barometers of climate change because of the rich database on migration contributed in large part by lay citizens [5,6,12,13]. Recent reviews of the large quantity of data leave little doubt of a fundamental impact of climate change on bird migration [14,15,16] although some of the specific mechanisms of migratory control and behavior have been recently challenged [17].

Over the past 18 years, I have been coordinating a citizen-science effort to document the first arrival dates of Maine migratory breeding birds across the state [18,19,20]. Previous comparisons of these modern data to data taken over a century ago from the Journal of the Maine Ornithological Society (published from 1899 until 1911) provide mixed evidence of earlier contemporary arrivals for some but not all species [21]. Over the eighteen-year period of the study, arrival dates are associated with fluctuations in mean springtime temperature as well as by the strength of the North Atlantic Oscillation (NAO) [19,20]. 

In this contribution, I take a longer view of the data. Rather than concentrating on the impact of various climatic variables on arrival dates, I explore the statistical distribution of arrival dates reported by all observers. As argued by [19], migratory birds are under competing pressures that result in stabilizing selection. On the one hand, birds, especially males, are being pushed to arrive as early as possible on the breeding grounds to stake out the most favorable breeding territories. On the other hand, birds are pressed to delay arrival in Maine until sufficient food is available to support them. We therefore expect that the distribution of arrival dates might yield a normal distribution. However, a positive or negative skew to the data could indicate that one of the two selective pressures is stronger than the other. I examine the distributions of arrival dates for each species and year for skewness and kurtosis. Kurtosis, a measure of the peakedness of a distribution, might be influenced by these selective pressures as well. A positive kurtosis (leptokurtosis) would indicate a prominent peak with particularly narrow tails, indicative of strong stabilizing selection. I ask if skewness and kurtosis are related to variation in springtime temperature, to the NAO, to foraging types of the birds, and to wintering area of the birds.

I also break down the distributions to see if the dates of arrivals of the earliest arriving individuals of a species can predict the arrival of the later arriving birds. I examine the interannual correlations of arrivals for 65 species of birds. With this deeper view, I am less focused on the responses of particular birds and more focused on discovering patterns by examining elements of the distributions of arrival dates. 

## 2. Materials and Methods

**Figure 1:**
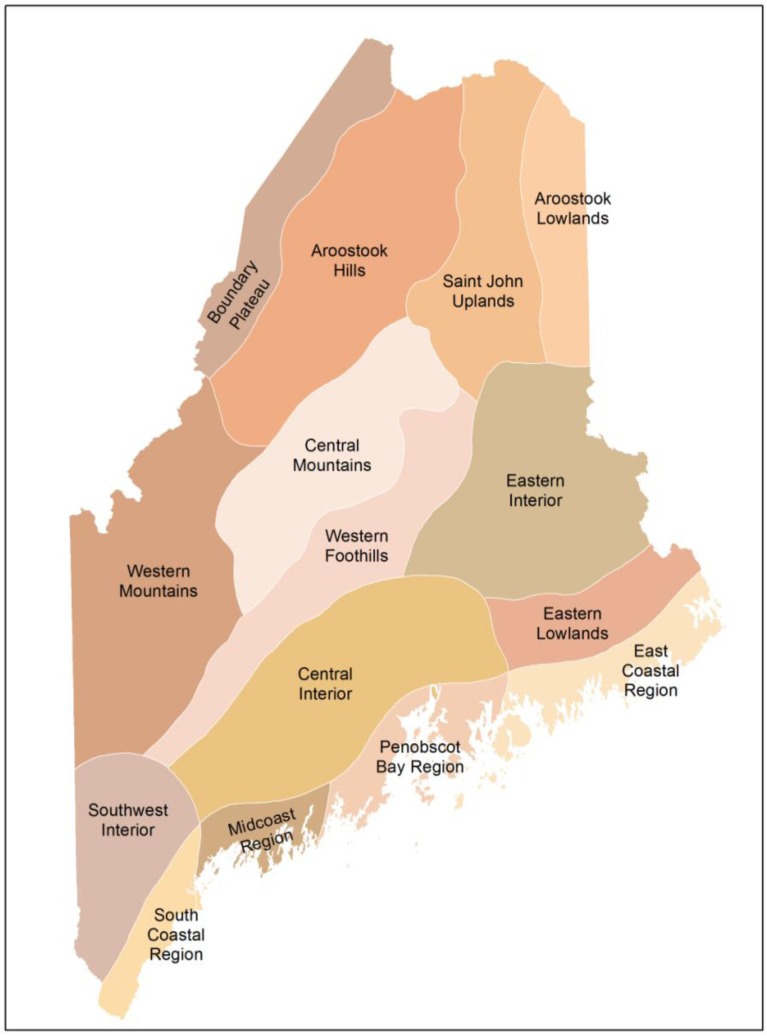
Map of Biophysical Regions of Maine, based on [22]. Map from the Maine Office of Geographic Information Systems (MEGIS).

The data for this study come from the 18 years (1994–2011) of data on arrival dates of Maine migratory breeding birds [18,19,20]. Each volunteer records the first arrival of over 100 species of migratory breeding bird and the Biophysical Region (Figure 1) in which each observation was made; [22] delineated these Biophysical Regions based on climate and vegetation patterns. Over 44,000 records have been reported by over 200 volunteers across the state. Although I have data on 145 species of migratory breeding birds, I restrict this study to an analysis of each of the 65 species that have a minimum of 20 arrival dates reported for each year of the study. For each spring, I calculated the departure of mean temperature (TempDep) from the 100-year mean. I obtained the 100-year mean by averaging data from 15 different meteorological stations across the state. I determined TempDep for March, April and May. The appropriate TempDep value was used for species that mostly arrive within a particular month. However, some species have arrival dates that span two months (e.g., late March into early April for Killdeer). For such species that arrive in late March into early April, I averaged the March and April TempDep values. Similarly, I averaged April and May for those species that arrive in late April into early May. I also used the NAO index for each year of the study.

Birds were categorized into one of nine foraging types (Table 1). Most of the foraging types are straightforward [23]. Gleaners take insects from leaves, granivores take seeds (mostly from the ground) while ground predators take animal prey from the ground. Scansorial predators take insects and other arthropods from the bark of trees.

**Table 1:** Foraging types, skewness, kurtosis and major migration period of the 65 species used in this study. The skewness and kurtosis values are the mean of the 18 yearly values. An asterisk after particular skewness or kurtosis values indicates that the value is significantly different from zero in Wilcoxon rank tests using the Bonferroni correction to insure an experiment-wise *p*-value of 0.05.

Species	Foraging Type	Skewness	Kurtosis	Migration Period
Ring-necked Duck (*Aythya collaris* (Donovan))	Aquatic	0.240	3.354 *	April
Great Blue Heron (*Ardea herodias* (Rackett))	Aquatic	0.620 *	4.667 *	April
Turkey Vulture (*Cathartes aura* (L.))	Scavenger	0.509 *	3.757 *	March/April
Osprey (*Pandion haliaetus* (L.))	Raptor	0.699 *	4.687 *	April
Broad-winged Hawk (*Buteo platypterus* Viellot))	Raptor	−0.390	4.249 *	April
American Kestrel (*Falco sparverius* L.)	Raptor	0.449 *	4.001 *	April
Killdeer (*Charadrius vociferus* L.)	Ground predator	0.873 *	4.392 *	March/April
Spotted Sandpiper (*Actitis macularius* (L.))	Ground predator	0.161	2.965 *	May
Wilson's Snipe (*Gallinago delicata* Ord)	Ground predator	0.476 *	2.861 *	April
American Woodcock (*Scoloplax minor* Gmelin)	Ground predator	1.231 *	5.490 *	March/April
Chimney Swift (*Chaetura pelagica* (L.))	Aerial	0.523	3.863 *	May
Ruby-throated Hummingbird (*Archilochus colubris* (L.))	Nectarivore	0.108	6.901 *	May
Belted Kingfisher (*Megaceryle torquata* (L.))	Aquatic	−0.636	5.357 *	April
Yellow-bellied Sapsucker (*Sphyrapicus varius* (L.))	Scansorial	1.479 *	3.578 *	April
Northern Flicker (*Colaptes auratus* (L.))	Scansorial	−0.783	7.253 *	April
Eastern Wood-Pewee (*C. virens* (L.))	Aerial	−0.729	6.373 *	May
Least Flycatcher (*E. minimus* (Baird and Baird))	Aerial	0.283	3.745 *	May
Eastern Phoebe (*Sayornis phoebe* (Latham))	Aerial	1.907 *	5.463 *	March/April
Great Crested Flycatcher (*Myiarchus crinitus* (L.))	Aerial	0.582	4.525 *	May
Eastern Kingbird (*Tyrannus tyrannus* (L.))	Aerial	−0.518	6.045 *	May
Blue-headed Vireo (*Vireo solitarius* (Wilson))	Leaf-gleaner	0.478	3.832 *	April/May
Warbling Vireo (*V. gilvus* Viellot)	Leaf-gleaner	0.533 *	2.883 *	May
Red-eyed Vireo (*V. olivaceus* (L.))	Leaf-gleaner	0.335	5.247 *	May
Tree Swallow (*Tachycineta bicolor* (Viellot)	Aerial	0.945 *	4.166 *	April
Barn Swallow (*Hirundo rustica* L.)	Aerial	0.173	3.037 *	May
House Wren (*Troglodytes aedon* Viellot)	Ground predator	0.277	3.239 *	May
Winter Wren (*Troglodytes troglodytes* L.)	Ground predator	0.627*	2.792*	April
Ruby-crowned Kinglet (*Regulus calendula* (L.))	Leaf-gleaner	0.454	3.725 *	April/May
Eastern Bluebird (*Sialia sialis* (L.))	Ground predator	0.225	3.708 *	April
Veery (*Catharus fuscescens* (Stephens))	Ground predator	−0.181	3.808 *	May
Hermit Thrush (*C. guttatus* (Pallas))	Ground predator	0.301	4.693 *	May
Wood Thrush (*Hylocichla mustelina* (Gmelin))	Ground predator	0.175	3.891 *	May
Gray Catbird (*Dumetella carolinensis* (L.))	Ground predator	−0.637	8.439 *	May
Brown Thrasher (*Toxostoma rufum* (L.))	Ground predator	0.217	3.579 *	May
Ovenbird (*Seiurus aurocapilla* (L.))	Leaf-gleaner	0.216	5.452 *	May
Northern Waterthrush (*Parkesia novaeboracensis* (Gmelin))	Leaf-gleaner	0.367	3.585 *	May
Black-and-white Warbler (*Mniotilta varia* (L.))	Leaf-gleaner	0.653	3.838 *	April/May
Nashville Warbler (*Oreothlypis ruficapilla* (Wilson))	Leaf-gleaner	0.804 *	4.546 *	May
Common Yellowthroat (*G. trichas* (L.))	Leaf-gleaner	0.928 *	5.056 *	May
American Redstart (*Setophaga ruticilla* (L.))	Leaf-gleaner	0.448	5.434 *	May
Northern Parula (*S. americana* (L.))	Leaf-gleaner	0.098	5.056 *	May
Magnolia Warbler (*S. magnolia* (Wilson))	Leaf-gleaner	0.262	3.562 *	May
Blackburnian Warbler (*S. fusca* (Forster))	Leaf-gleaner	0.628	4.514 *	May
Yellow Warbler (*S. petechia* (L.))	Leaf-gleaner	0.470	4.187 *	May
Chestnut-sided Warbler *(S. pensylvanica* (L.))	Leaf-gleaner	0.629	4.823 *	May
Black-throated Blue Warbler (*S. caerulescens* (Gmelin))	Leaf-gleaner	0.652 *	3.838 *	May
Palm Warbler (*S. palmarum* (Wilson))	Leaf-gleaner	0.579	3.824 *	April
Pine Warbler(*S. pinus* (Wilson))	Leaf-gleaner	0.651	4.208 *	April
Yellow-rumped Warbler (*S. coronata* (L.))	Leaf-gleaner	−0.835	8.506 *	April/May
Black-throated Green Warbler (*S. virens* (Gmelin))	Leaf-gleaner	0.667 *	4.061 *	May
Canada Warbler (*Cardellina canadensis* (L.))	Leaf-gleaner	0.320	3.429 *	May
Chipping Sparrow (*Spizella passerina* (Bechstein))	Granivore	−0.254	4.446 *	April
Savannah Sparrow (*Passerculus sandwichensis* (Gmelin))	Granivore	0.014	3.676 *	April
Song Sparrow (*Melospiza melodia* (Wilson))	Granivore	−0.792	8.560 *	Mar/April
Swamp Sparrow (*M. georgiana* (Latham))	Granivore	0.094	3.426 *	April
White-throated Sparrow (*Zonotrichia albicollis* (Gmelin))	Granivore	−1.986 *	7.842 *	April
Scarlet Tanager (*Piranga olivacea* (Gmelin))	Leaf-gleaner	0.651 *	3.746 *	May
Rose-breasted Grosbeak (*Pheucticus ludovicianus* (L.))	Granivore	0.764	6.092 *	May
Indigo Bunting (*Passerina cyanea* (L.))	Granivore	0.157	3.026 *	May
Bobolink (*Dolichonyx oryzivorus* (L.))	Ground predator	0.605	7.210 *	May
Red-winged Blackbird (*Aegelaius phoeniceus* (Müller))	Ground predator	1.483 *	6.222 *	Mar
Eastern Meadowlark (*Sturnella magna* (L.))	Ground predator	1.992	2.583 *	April
Common Grackle (*Quisculus quiscala* (L.))	Ground predator	1.426 *	7.179 *	Mar
Baltimore Oriole (*Icterus galbula* (L.))	Leaf-gleaner	0.010	7.857 *	May

I classified each of the 65 species according to its major wintering area: South America, Central America, Caribbean, or southeastern United States. Central and South America along with Caribbean were combined into a Long category for the long-distance migration those birds undertake. Long contrasts with Short, referring to those birds wintering in the United States with a less arduous migration.

For each species-year combination, I used Stata 12 statistical software to calculate the skewness and kurtosis [24]. Because skewness and kurtosis data are not normally distributed, non-parametric tests were used to test for differences among factors. I used the Wilcoxon signed-rank test to determine if the skewness and kurtosis for each species differed from a normal distribution by testing the observed values against a value of 0 (the value of skewness and kurtosis in a normal distribution). For both the skewness and kurtosis analyses, I used the Bonferroni correction to minimize Type I errors from performing multiple analyses [24]. The correction was set to ensure that an experiment-wise value of *p* = 0.05 was obtained by setting the critical *p*-value for each test as 0.0007, obtained by dividing 0.05 by 65 (the number of tests performed). Using the non-parametric Kruskall-Wallis rank test, I examined the relationship between arrival month, foraging type, migration distance and wintering area on both kurtosis and skewness. I used the Bonferroni correction for these four tests, setting the critical *p*-value of each test to *p* = 0.0125, yielding an experiment-wise critical *p*-value of 0.05. I used linear regression analysis to examine the ability of TempDep and NAO index to predict kurtosis and skewness. The critical *p*-value of 0.0007 was used here as well to produce an experiment-wise critical value of *p* = 0.05.

For the distribution of each species-year combination, I calculated five metrics: the 5% median, the 10% median, the 25% median, the true median (50% median) and the 75% median. I used Pearson product-moment correlations to evaluate the pair-wise relationship between each type of median across the 18 years of the study.

Using the values of the 25% median, 50% median and 75% median, I used Pearson correlation tests to test for significant relationships between pairs of species that were migrating in the same time interval. Thus, I developed one table of all possible combinations of species arriving in a particular month. This analysis tests for the independence of migration schedules among the species in the study.

## 3. Results

The quantity of arrival data for each Biophysical Region (Figure 1) in this study reflects the human population density. The most observations came from the Central Interior (31.6% of all observations), the South Coastal Region (21.8%), the Midcoast Region (17.3%), the Penobscot Bay Region (8.2%) and the East Coastal Region (7.9%). Most of the remaining data (13.2%) were spread equally across the Southwest Interior, the Western Foothills, the Eastern Lowlands and the Eastern Interior. 

**Figure 2:**
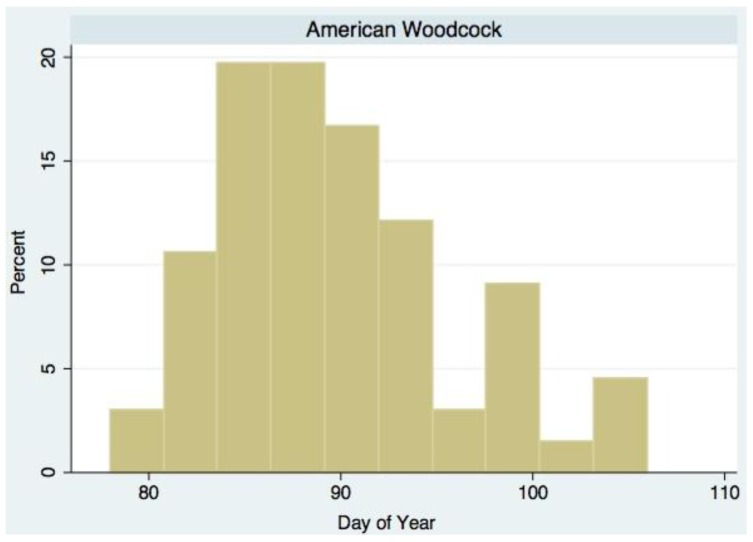
Distribution of first arrival dates for American Woodcock for 1994. The distribution is right-skewed with some leptokurtosis (more pronounced peak and thinner tails than seen in a normal distribution).

Table 1 presents the mean values of skewness and kurtosis for the 65 species examined in this paper. In a symmetrical distribution, skewness would be equal to zero. Fifty-four of the 65 species have a positive value of skewness. Using Wilcoxon signed-rank tests, 20 of the species show skewness values that are significantly different from zero. White-throated Sparrow is the only species that has a statistically significant negative skewness. Figure 2 presents the distribution of arrival dates of American Woodcock for 1994 as a typical example. This distribution has a positive skewness with the longer tail to the right.

Wilcoxon signed-rank tests indicate every species has a significant positive kurtosis (a value of zero is expected for a normal distribution). The positive kurtosis is termed leptokurtosis, indicating a pronounced peak in the middle of the distribution with thinner than expected tails to the distribution.

I used a Kruskal-Wallis rank test to determine if foraging type (Table 2) was associated with the degree of skewness. The differences were not significant (**Χ**^2^ = 21.81, *p* = 0.01). Scavengers and leaf-gleaners had the highest positive skewness and raptors had the lowest value. Similarly, a Kruskal-Wallis rank test, examining the effect of foraging type on kurtosis was also significant (**Χ**^2^ = 65.64, *p* < 0.01). Nectarivores were particularly leptokurtic with aquatic feeders and scavengers showing less pronounced leptokurtosis.

**Table 2:** Relationship of foraging type on kurtosis. A Kruskal-Wallis rank test indicated significant differences among feeding types.

Foraging type	Skewness	Kurtosis
Aerial insectivore	0.38	4.65
Aquatic feeder	0.35	3.09
Gleaner	0.45	4.59
Granivore	0.32	5.48
Ground predator	0.31	4.41
Nectarivore	0.06	7.38
Piscivore	0.22	4.13
Raptor	0.03	4.90
Scansorial predator	0.35	5.42
Scavenger	0.51	3.76

A Kruskal-Wallis rank test was used to test the statistical effect of migration month on skewness and kurtosis (Table 3). A significant effect was found for skewness (**Χ**^2^ = 49.70, *p* < 0.01) and kurtosis (**Χ**^2^ = 30.43, *p* < 0.01). Early arriving species (Red-winged Blackbird and Common Grackle) were associated with the highest skewness and highest kurtosis. The May arrivals showed a slight negative skew.

**Table 3:** The relationship of migration period on skewness and kurtosis. Means are provided. Migration period had significant effects on both measurements in Kruskal-Wallis rank tests.

Migration period	Skewness	Kurtosis
March	1.45	6.73
March/April	0.57	4.65
April	0.26	4.38
April/May	0.24	4.57
May	−0.29	4.83

Kruskal-Wallis rank tests failed to show an effect of wintering area (and hence migratory distance) on skewness (**Χ**^2^ = 7.34, *p* = 0.02) and on kurtosis (**Χ**^2^ = 3.32, *p* = 0.65). Combining the three tropical regions into one region to contrast with the US region fails to show a significant effect as well (**Χ**^2^ = 0.37, *p* = 0.55 for skewness and **Χ**^2^ = 0.21, *p* = 0.35 for kurtosis).

To examine the statistical effect of TempDep on skewness, I used regression analyses. With the Bonferroni correction, no significant relationship was found any of the 65 species (all *p*-values > 0.0007). Similarly, regression analyses were used to test the statistical effect of TempDep on kurtosis. None of the 65 regressions was significant (*p* > 0.0007 in all cases).

No significant relationship of NAO index with skewness was detected by regression analysis for all 65 species. Regression analyses also failed to find a relationship between NAO index and kurtosis. None of these 130 regressions yielded a p-value below the Bonferroni-corrected value of *p* = 0.0007.

Table 4 provides a summary of the correlation of different portions of the distribution (5% median, 10% median, 25% median, 50% median and 75% median). Percentages represent the fraction of species showing a significant relationship. For instance, for the 10 March/April migrants, the 5% median and 10% median are significantly correlated for all 10 species but only five of the 5% median to 25% median correlations are significant. A consistent pattern throughout is that adjacent medians are much more likely to be correlated than medians from different parts of the distribution (5% median correlated with the 75% median, for example).

Table 4Percentage of species whose various medians show a significant Pearson correlation (*p* < 0.05).

**Table:** March–April (10 species)

	5%	10%	25%	50%
10%	100			
25%	50	80		
50%	20	50	10	
75%	20	30	40	100

**Table:** April (16 species)

	5%	10%	25%	50%
10%	88			
25%	38	81		
50%	25%	43	88	
75%	25	31	44	69

**Table:** April–May (6 species)

	5%	10%	25%	50%
10%	83			
25%	50	67		
50%	17	17	83	
75%	0	0	17	67

**Table:** May (31 species)

	5%	10%	25%	50%
10%	87			
25%	58	84		
50%	26	55	77	
75%	6	16	32	94

**Figure 3:**
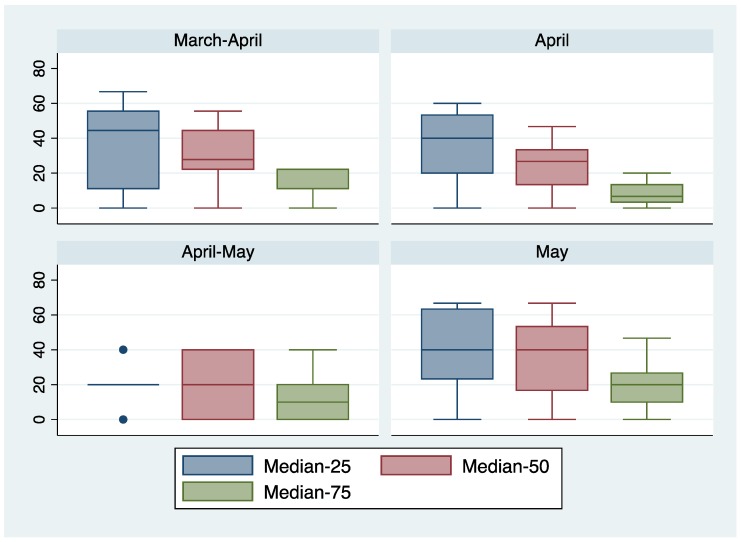
Box plots showing the percentage of interspecific significant correlations (*p* < 0.05) using the 25% median, 50% median and 75% median. Data for March are not presented because only two species arrive then (Red-winged Blackbird and Common Grackle). The box plot for April–May is based on only six species, accounting for its collapsed appearance.

Figure 3 presents a summary of the interspecific correlation analyses. The 25% median, 50% median and 75% median were analyzed separately. Each species migrating in a particular month was analyzed with every other species for that month. A consistent pattern is that the percentage of significant correlations for the 75% median is always the lowest. For three of the four time intervals, the 25% median had the highest percentage of significant correlations. For the April–May interval, only six species were analyzed making general patterns harder to discern.

## 4. Discussion

To seek effects of climate change on bird migration, ornithologists have used two sorts of data. A few studies have been conducted where the arrival of all members of a local population of a few species are recorded through a banding program or intensive censusing of territorial males [25,26]. This total population approach provides ideal data but the effort required is enormous. Instead, most of the data collected to examine the impact of global climate change on birds come from first arrival dates [13,14,15,17,18,19,20,21]. Such data are easily obtained and the contribution of amateurs to this database is significant. The disadvantage is that we are only sampling the left-hand portion of the distribution, the vanguard of the migratory wave [17].

The second approach was used in this study. The analysis in this contribution was designed to seek patterns of deviation from the normal distribution in the shape of the arrival date curves. Studies using arrival dates of migratory birds to seek evidence of global climate change have concentrated on the mean or median dates [2,5,6,8,9,11]. I argue that the shape of the distribution of arrival dates may offer additional insight. Patterns of skewness and kurtosis have been profitably quantified in studies using the total population approach [25,26].

The first result from this analysis is that the distributions of arrival dates have third (skewness) and fourth (kurtosis) moments that differ from a normal distribution. In the majority of cases, the distribution shows a right skew (Figure 2, Table 1). The distributions also show leptokurtosis, a more pronounced central peak and thinner tails, than would be seen in a normal distribution (Figure 2, Table 1).

I believe these patterns of skewness and kurtosis are informative in relation to the selective pressures on arrival date. The piling up of values on the left side of the distribution implies that the pressure to delay migration until food resources are available is stronger than the pressure to arrive early to procure a favorable territory. The leptokurtic distribution suggests that the median arrival dates are even more advantageous than one would expect if the data were normally distributed.

Foraging type of birds had an impact on the skewness of yearly arrival date distributions (Table 2). I expected that species whose food is more seasonal (herbivorous insects for leaf-gleaners, nectar for nectarivores) would show stronger right skew than raptors. That pattern was observed. I also found a significant relationship between foraging type and kurtosis. Highest kurtosis values were found for nectarivores, suggesting that there is a narrow optimal arrival window for these species.

The month of arrival was correlated with skewness and kurtosis values. Arrivals in March are more perilous than later arrivals because of the fickle nature of the Maine spring. One predicts more of a right-skewed and leptokurtic distribution for early arrivals. That pattern was observed with the two earliest migrants, Red-winged Blackbird and Common Grackle, showing highest skewness and kurtosis [27] presented data to show that long-distance migrants are showing a stronger effect to global climate change than short-distance migrants (although see [19]). In New York and Massachusetts, Neotropical migrants are arriving about 13 days earlier on average now compared to 50 years ago while short-distance migrants are arriving only four days earlier. Insofar as a wintering bird in South America cannot predict the weather in the northern United States, we might expect such species to show less pronounced skewness and kurtosis than intracontinental migrants that can time their migration to regional temperature regimes. Surprisingly, wintering area did not have any statistical effect on either skewness or kurtosis.

For all species, the skewness of yearly distributions of arrival dates was not related to the deviation of springtime temperatures from the long-time average. Temperature patterns occur on a broad-scale from Delaware north through Maine [19], allowing northward migrants to predict springtime conditions 500 km or more to the north. One might expect migratory birds to exercise caution when springs are colder than normal. I am surprised that none of the 65 species showed this type of response. Similarly, none of the species showed a response of yearly kurtosis values to deviation from normal temperatures.

The larger scale driver of weather, the North Atlantic Oscillation, is related to the timing of bird migration [19,28,29]. However, the NAO had no statistically significant relationship with skewness and kurtosis for any of the 65 species. 

We have abundant evidence now that both photoperiod and weather patterns influence the timing of avian migration [30,31,32,33]. Breaking down the distributions of arrival dates for each year yields insight into the strength of these two cues. As shown in Table 2, stronger correlations consistently exist for adjacent portions of the distribution (e.g., the 5% median with the 10% median) than for portions of the distribution more widely separated (e.g., the 10% median with the 75% median). I interpret this consistent result as evidence for the primacy of weather-temperature responses to the migration. If photoperiod were driving the migration, we would expect more consistent correlations among portions of the distribution more distantly removed.

Figure 3 explores the relationship of arrival dates between species that arrive during the same time period (March–April, April, April–May, May). Because many of the species arriving in the same month have similar dietary requirements, correlations of arrival dates are not surprising. The unexpected result is the consistently higher proportion of significant correlations between species for earlier migrant. The proportion of significant correlations is greatest for the 25% median and least for the 75% median. This result is consistent with the general pattern (Table 1) of a right skew of the distributions of arrival dates. The correlation data and the skewness data suggest that a greater penalty ensues for arriving too early than for arriving too late.

## 5. Conclusions

In this paper, I have taken a longer, exploratory view of an 18-year dataset of arrival dates for 65 species of migratory birds that nest in Maine. Right-skewed arrival dates (Table 1) indicate that there is greater selection pressure against arriving too early than arriving later. Leptokurtic patterns suggest strong selection for the optimal arrival date. Tighter correlations between the arrival of species at the vanguard of migration (the 25% of median) compared to later groups of arrivals supports the notion that selection is greatest for the earliest arrivals. Breaking the distribution of arrivals into different medians indicates that fine-tuning of the wave of migration occurs as individuals of a species migrate into Maine (Table 2).
